# Beyond speed and strategy: A configurational analysis of collective attention to post-match institutional discourse in the UEFA Champions League

**DOI:** 10.1371/journal.pone.0354175

**Published:** 2026-07-28

**Authors:** Zhichen Ling, Xinlei Ren, Bingquan Luo

**Affiliations:** School of Management and Communication, Capital University of Physical Education and Sports, Beijing, China; Universiti Malaya, MALAYSIA

## Abstract

Post-match institutional communication has become a visible component of elite football events, yet comparable official messages can receive very different levels of public attention. This study examines UEFA Champions League post-match communication as a configurational problem rather than as a linear consequence of response speed or message strategy. Using 25 theoretically selected focal club-event observations from the 2017–2018–2022–2023 seasons, it calibrates one outcome set, High_News, from a Raw_Mentions-derived media-visibility proxy and four causal-condition sets: High_Delay, High_Shock, High_Stakes, and High_TemplateDeviation. The analysis uses conservative coding of template deviation, separating response latency from communication form. The intermediate solution identifies one stable high-stakes legacy pathway and one sensitivity-dependent crisis-minimalism pathway. The stable pathway combines non-delayed response, low outcome shock, high match stakes, and trophy/legacy-oriented template deviation. The crisis-minimalism pathway is retained in the baseline solution but is interpreted cautiously because it depends on medium-confidence coding and changes under strict recoding. The analysis of ~High_News confirms causal asymmetry: non-membership in the high-attention set is not simply the inverse of the pathway(s) associated with High_News. The findings contribute to sport communication research by showing how event structure and institutional discourse combine to produce bounded media visibility, while avoiding population-level claims about all football matches or internal platform algorithms.

## 1. Introduction

Elite football clubs now communicate in an attention environment where sport performance, platform affordances, journalistic uptake, and fan affect interact. A post-match message issued through an official club account is not merely a notice of a result. It is an institutional act that can be searched, quoted, reframed, circulated, and contested. This creates a recurrent empirical puzzle: some routine-looking messages remain peripheral, whereas other messages with similar surface form become focal points for news coverage, memes, reputational commentary, and collective memory.

Existing research provides useful but incomplete explanations for this puzzle. Sport communication scholarship has shown that digital platforms have altered the relationship between organizations, journalists, athletes, and fans [1,2]. Crisis-communication research has further shown that organizational responses are evaluated through responsibility, timing, and message strategy [3,4]. These perspectives explain why response timing and message fit matter. They are less able to explain why the same communicative attribute may have different outcomes depending on match stakes, outcome shock, and the public meaning of the event.

The present study reframes post-match attention as a set-theoretic outcome. Rather than asking whether response delay, match stakes, outcome shock, or discursive form has an isolated effect, it asks which combinations of conditions are associated with membership in the set of high collective attention. This formulation follows configurational theory, which treats conjunctural causation, equifinality, and causal asymmetry as empirical features of social phenomena [5–8]. It is also substantively suited to elite sport, where attention rarely follows from organizational communication alone and instead emerges from the encounter between event structure and institutional discourse.

Empirically, the study examines 25 focal club-event observations from UEFA Champions League matches between the 2017–2018 and 2022–2023 seasons. The contribution is methodological and substantive. Methodologically, the study provides a transparent small-N fsQCA design with a case-selection audit, a reproducible Raw_Mentions-derived outcome proxy, truth-table reporting, robustness checks, and reproduction code. Substantively, the study shows that response speed is better understood as a threshold condition for entering the post-match visibility window, whereas the broader attention outcome depends on how timing, event shock, match stakes, and communication form combine.

## 2. Literature review and theoretical framework

### 2.1. Sport communication in a platformized attention environment

Sport communication has moved from a broadcast-dominant system toward a networked media environment in which clubs, athletes, journalists, and fans jointly shape visibility [1,2]. In this environment, official messages are not stable endpoints of communication. They become platform objects that may be searched, quoted, reframed, and redistributed. Platform studies indicate that visibility is governed by interactional and algorithmic logics rather than by organizational intention alone [9–12]. For elite sport organizations, this means that the consequences of a post are partly determined after it enters a contested attention field. Institutional pressures may also encourage organizations within the same field to adopt standardized and recognizable communication forms [13].

This point is especially relevant after high-intensity matches. Football events generate immediate affective publics: supporters and opponents evaluate not only the score but also the symbolic meaning of victory, collapse, humiliation, redemption, or legacy. A club message that appears institutionally routine may be treated by users as irony, solidarity, evidence, ridicule, or collective memory. The object of analysis here is therefore not sentiment alone but collective attention: whether a post-match institutional act becomes visible in wider news and search environments.

### 2.2. Limits of linear crisis-communication assumptions

Situational Crisis Communication Theory remains central to organizational crisis communication because it links perceived responsibility, reputational threat, and response strategy [3]. Sport crisis-communication research has also shown that fans act as crisis communicators rather than passive audiences [4]. These perspectives help explain why timing and response fit matter. Evidence from proactive crisis disclosure further indicates that early organizational self-disclosure can enhance perceived credibility [14]. Yet post-match communication in elite football differs from many organizational crises because the club is not always managing a discrete scandal; it is responding to a competitive event whose meaning has already been partly produced by the match.

The implication is that communication strategy should be examined conditionally. A rapid score post after a routine match may disappear quickly. The same rapid post after a historic collapse may become highly visible because the event supplies the primary affective force. Likewise, template deviation may align with celebration in one case and become irrelevant or risky in another. This conditionality weakens the explanatory value of isolated net effects and supports a configurational approach.

### 2.3. Configurational causation and set-theoretic attention

fsQCA is appropriate when the research problem involves conjunctural causation, equifinality, and causal asymmetry [5–8,15,16]. In a set-theoretic design, cases have degrees of membership in calibrated sets. The question is not whether an independent variable increases a dependent variable on average, but whether a condition or combination of conditions is consistently associated with membership in an outcome set. In this study, High_News denotes membership in the set of high collective attention cases. The negated outcome, ~ High_News, denotes non-membership in that set, not a separate linear variable called low attention.

**Fig 1:**
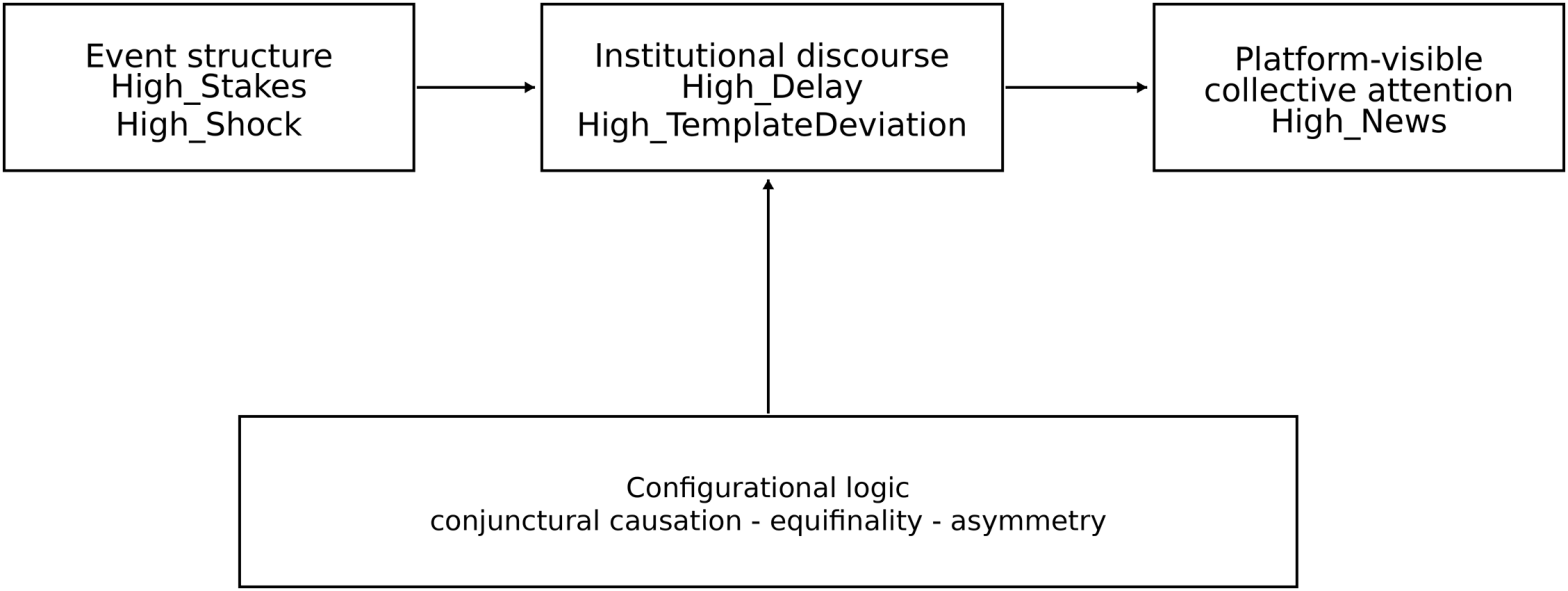
Configurational framework linking event structure, institutional discourse, platform-visible collective attention, and set-theoretic analysis.

The theoretical expectation is therefore conditional. High collective attention should not be attributed to speed, shock, stakes, or template deviation in isolation. It should emerge when event structure and communication form are configured in a way that allows an official message to enter and remain visible in the immediate post-match attention window. Fig 1 summarizes this logic.

## 3. Materials and methods

### 3.1. Case selection and case boundary

The case boundary was defined as UEFA Champions League post-match events from the 2017–2018–2022–2023 seasons for which a relevant club-issued post-match message and external attention indicators were available. A case was defined as one club’s first substantive official post-match communication following a Champions League match that generated a salient sporting outcome. The unit of analysis is therefore the club-event-message, not the match alone. The final sample includes 25 focal club-event observations.

The study uses explicit theoretical-sampling criteria rather than relying on the undefined label “iconic.” A case was eligible when it met at least two of the following criteria: final or knockout-stage structural salience; a clearly identifiable title, humiliation, reversal, miracle, or final-loss narrative; available timing for the focal club’s first substantive institutional post; and retrievable Raw_Mentions within the post-match attention window. The case-selection audit in S1 File reports retained and non-retained candidates and explains the inclusion or exclusion decision. This sampling logic is appropriate for a small-N fsQCA design but is not statistically representative of all Champions League matches.

### 3.2. Data sources and measurement window

The outcome set, High_News, is based on a reproducible media-visibility proxy rather than on a broader opaque composite index. Raw_Mentions records the retrievable post-match media mentions associated with the focal club-event observation within the immediate post-match window. News_Index is generated by applying a natural-log transformation to Raw_Mentions and then normalizing the result to a 0–100 scale. Because platform-level search-intensity data were not consistently available for all historical cases, News_Index is not treated as a comprehensive public-attention index or as a weighted composite of news volume and search intensity.

Response timing is measured as Raw_Delay, the number of minutes between the final whistle and the focal club’s first substantive post-match communication. High_Shock is assigned from the event narrative and separates outcome disruption from communication form. High_Stakes is assigned from tournament stage. High_TemplateDeviation is assigned only from the audited Deviation_Type field. It is not inferred from Raw_Delay, the match result, or the event trigger. This separation was introduced to avoid circularity between response latency and communication form.

### 3.3. Calibration strategy

For quantitative variables, membership scores follow direct piecewise linear calibration. Values at or above the full-membership anchor receive 1.0; values at or below the full-non-membership anchor receive 0.0; values between the crossover and either endpoint are linearly interpolated. High_Shock, High_Stakes, and High_TemplateDeviation are fuzzy-set assignments based on theoretically specified categories rather than interval-scale measurements. The full case-level coding is provided in S1 File.

### 3.4. Template-deviation audit

High_TemplateDeviation required the most conservative audit. A less conservative specification would risk treating delayed silence, rapid post-loss communication, or a dramatic result itself as evidence of template deviation. The specification is conservative. High_TemplateDeviation is assigned only when the observable form of the focal communication departs from routine result reporting through trophy or legacy framing, unusually heightened affective celebration, symbolic non-score-led framing, or crisis-minimalist reporting after an extreme defeat. Cases that depended on response delay or rapid post-loss timing are coded as Standard_Report in the main specification or moved to robustness checks.

The study uses a structured author audit rather than an intercoder-reliability design; no intercoder reliability coefficient is claimed. To make this limitation visible rather than hidden, the analytical dataset in S1 File reports Post_URL when available, Post_Text_Excerpt_or_Audit_Note, Source_Access_Note, Deviation_Type, Deviation_Evidence, Coding_Confidence, and Robustness_Flag for each case. The strict robustness checks then test whether coding-sensitive cases alter the solution.

### 3.5. Analytical procedure

**Fig 2:**
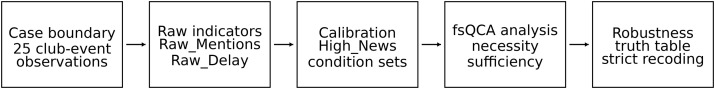
Research design and analytical workflow. All reported values are recalculated from the analytical dataset using the reproduction code provided in S1 File.

The analysis proceeded in five steps. First, the 25-case analytical dataset was reconstructed and aligned with the case-selection audit. Second, all sets were calibrated from the analytical dataset in S1 File using the rules reported in Table 1. Third, necessity analysis assessed whether any single condition reached the conventional 0.90 consistency benchmark. Fourth, sufficiency analysis examined observed truth-table rows for membership in High_News. Fifth, robustness checks tested alternative calibration anchors, stricter evidence rules, conservative template recoding, and an alternative structural-silence specification. The case-selection audit, observed truth-table rows, robustness checks, and reproduction code are provided in S1 File (Fig 2).

**Table 1:** Operationalization and calibration of fuzzy-set conditions.

Set	Operational meaning	Full membership	Crossover	Full non-membership	Robustness strategy
High_News	Membership in high collective attention	News_Index >= 90	News_Index = 60	News_Index <= 30	Alternative anchors 85/55/25 and 95/65/35
High_Delay	Temporal delay of first substantive institutional post	Raw_Delay >= 360 minutes	Raw_Delay = 60 minutes	Raw_Delay <= 15 minutes	Alternative full-membership anchors 300 and 420 minutes
High_Shock	Affective disruption generated by match outcome	Extreme humiliation/miracle/reversal	Moderate disruption	Routine or expected outcome	Category assignment provided in S1 File
High_Stakes	Structural importance of the fixture	Final	Quarter-final/semi-final continuum	Lower-stakes or early-stage equivalent	Stage assignments provided in S1 File
High_TemplateDeviation	Observable departure from routine result-reporting form	Trophy/legacy framing; affective celebration; symbolic non-score framing; crisis minimalism	Moderate, evidence-dependent departure	Standard score/result reporting or insufficient evidence	Strict-evidence and conservative-recoding checks in S1 File

## 4. Results

### 4.1. Necessary conditions

Necessity analysis indicates that no single condition, by itself, explains membership in High_News. Using the conventional 0.90 consistency benchmark, ~ High_Delay (consistency = 0.971) and High_Stakes (consistency = 0.928) meet the formal criterion for weak necessity, but their coverage values are modest. High_TemplateDeviation does not meet the necessity threshold after the conservative audit (consistency = 0.417), which means that template deviation should not be interpreted as a universal prerequisite for high collective attention. Table 2 reports the necessity results.

**Table 2:** Analysis of necessary conditions for membership in High_News.

Condition	High_News_consistency	High_News_coverage
High_Delay	0.103	0.552
~High_Delay	0.971	0.576
High_Shock	0.569	0.554
~High_Shock	0.523	0.617
High_Stakes	0.928	0.659
~High_Stakes	0.191	0.411
High_TemplateDeviation	0.417	0.927
~High_TemplateDeviation	0.583	0.41

### 4.2. Sufficiency and configurational pathways to High_News

**Fig 3:**
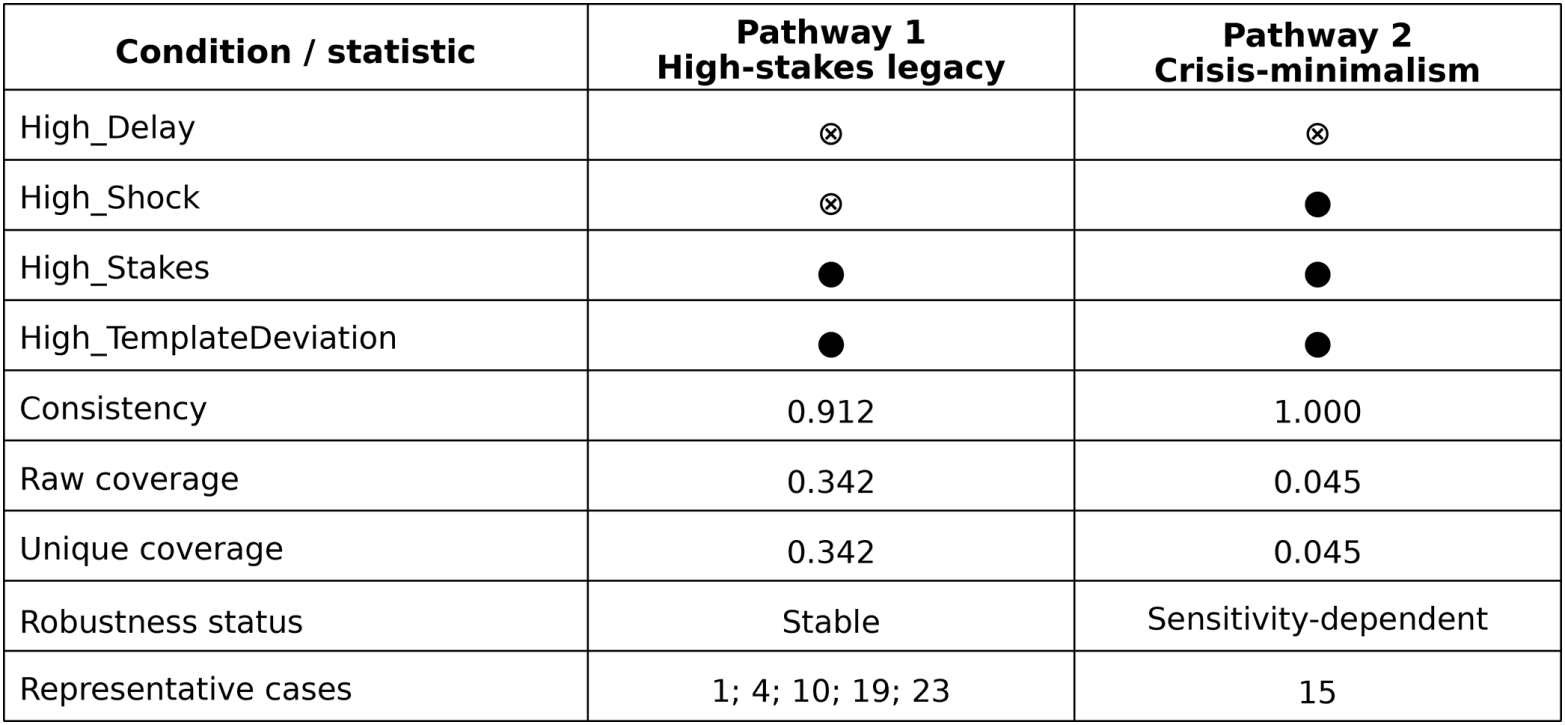
Configurational pathways to membership in High_News. The figure reports the intermediate fsQCA solution using standard configurational notation. ● indicates presence, ⊗ indicates absence, and blank cells would indicate conditions not specified. The sensitivity-dependent pathway is retained in the baseline solution but interpreted cautiously because it changes under stricter evidence and conservative coding checks.

The intermediate solution identifies one stable pathway and one sensitivity-dependent pathway associated with membership in High_News (Table 3). The stable pathway is ~High_Delay x ~High_Shock x High_Stakes x High_TemplateDeviation - > High_News (consistency = 0.912; raw coverage = 0.342). It is supported mainly by title-winning or legacy-framing cases: Man City 2023, Liverpool 2019, Real Madrid 2022, Bayern 2020, and Chelsea 2021. The second pathway is ~High_Delay x High_Shock x High_Stakes x High_TemplateDeviation - > High_News (consistency = 1.000; raw coverage = 0.045). It is represented by the Barcelona-Bayern 2020 humiliation case and is therefore interpreted as sensitivity-dependent rather than as a general crisis pathway. The overall solution consistency is 0.921, and overall coverage is 0.387 (Fig 3).

**Table 3:** Intermediate solution for membership in High_News.

Configuration	Formula (TD = High_TemplateDeviation)	Consistency	Raw coverage	Unique coverage	Representative cases
Stable high-stakes legacy pathway	¬Delay × ¬Shock × Stakes × TD	0.912	0.342	0.342	1; 4; 10; 19; 23
Sensitivity-dependent crisis-minimalism pathway	¬Delay × Shock × Stakes × TD	1.000	0.045	0.045	15
Overall solution	Union	0.921	0.387	–	1; 4; 10; 15; 19; 23

### 4.3. Case-based interpretation

The stable pathway shows that the strongest evidence concerns high-stakes title or legacy communication rather than template deviation in general. In these cases, the club responds without substantial delay, the outcome is not coded as high shock because the focal club wins the title, and the official message departs from neutral result reporting through ceremonial or legacy-oriented framing. This pathway has high consistency but moderate coverage. It should therefore be read as a bounded sufficient configuration, not as a comprehensive explanation of all high-attention cases.

The sensitivity-dependent pathway captures a different pattern: rapid communication after a high-shock, high-stakes defeat accompanied by crisis-minimalist form. Because this pathway is represented by a single medium-confidence case, the analysis does not treat it as a central finding. Its value is diagnostic: it shows why crisis-minimalist communication should be investigated in larger, text-richer samples, but it should not be generalized from the present dataset.

### 4.4. Non-membership in High_News

The analysis of ~High_News confirms causal asymmetry. Non-membership in High_News is not the simple inverse of the stable high-attention pathway. Several cases combine high stakes or high shock with non-membership in High_News because they lack the specific conjunction of non-delayed response and strong, independently coded template deviation. This finding supports the set-theoretic interpretation: absence of high attention cannot be inferred by reversing the conditions associated with high attention.

### 4.5. Robustness checks

Robustness checks are provided in S1 File. The stable high-stakes legacy pathway remains substantively similar under alternative High_Delay anchors, alternative High_News anchors, and strict evidence checks. The crisis-minimalism pathway is more fragile. When medium-confidence High_TemplateDeviation cases are conservatively recoded, or cases marked Recode_if_strict are treated as Standard_Report, the crisis-minimalism pathway is removed while the stable legacy pathway remains. This pattern strengthens the central interpretation by showing which configuration carries the main explanatory weight and which configuration should be treated cautiously (Table 4).

**Table 4:** Robustness checks for calibration and coding sensitivity.

Robustness_ID	Robustness_Test	Solution_Change	Interpretation
R1	Baseline model	stable high-stakes legacy pathway; sensitivity-dependent crisis-minimalism pathway	Main interpretation rests on the stable pathway; crisis-minimalism is sensitivity-dependent.
R2	Alternative High_Delay anchor	stable high-stakes legacy pathway; sensitivity-dependent crisis-minimalism pathway	Stable pathway remains the main solution under a less restrictive delay full-membership anchor.
R3	Alternative High_Delay anchor	stable high-stakes legacy pathway; sensitivity-dependent crisis-minimalism pathway	Stable pathway remains the main solution under a more restrictive delay full-membership anchor.
R4	Alternative High_News anchors	stable high-stakes legacy pathway; sensitivity-dependent crisis-minimalism pathway	The solution is not produced by a single outcome threshold, but coverage changes as expected.
R5	Alternative High_News anchors	stable high-stakes legacy pathway; sensitivity-dependent crisis-minimalism pathway	Stricter outcome calibration narrows coverage; interpretation remains conservative.
R6	Strict evidence model	stable high-stakes legacy pathway; sensitivity-dependent crisis-minimalism pathway	The stable pathway remains interpretable because it is driven by high-confidence title/legacy cases.
R7	Conservative template recoding	stable high-stakes legacy pathway	The crisis-minimalism pathway is removed; the legacy pathway remains the main finding.
R8	Recode-if-strict model	stable high-stakes legacy pathway	The stable pathway remains; disputed loss-response cases do not drive the main result.
R9	Structural-silence alternative	stable high-stakes legacy pathway; sensitivity-dependent crisis-minimalism pathway	Structural silence is not part of the main model and does not change the main legacy interpretation.

The detailed consistency and coverage values for each robustness check are provided in S1 File.

## 5. Discussion

### 5.1. What the configuration explains

The analysis narrows the interpretation of High_TemplateDeviation. Rather than treating every high-shock or delayed-response case as a departure from routine communication, the conservative audit identifies the most empirically defensible form of template deviation in this sample: trophy or legacy-oriented post-match framing in high-stakes contexts. This narrower result is methodologically preferable because it separates event shock, response delay, and communication form.

The main finding is therefore not that template deviation universally increases attention. It is that a particular configuration – non-delayed response, high stakes, low outcome shock for the focal club, and strong title/legacy framing – is consistently associated with membership in High_News. This interpretation is conservative and methodologically stronger because it avoids deriving communication form from match result or timing.

### 5.2. Conditional visibility and sport communication theory

The findings refine crisis-communication assumptions in elite sport. SCCT-oriented thinking explains why timing and response fit matter under reputational pressure [3]. The results show that response fit is conditional on event structure and public affect. Rapid response places a club inside the post-match visibility window, but visibility becomes stronger only when rapid entry is configured with high stakes and a communicative form that audiences and news systems can recognize as historically or symbolically meaningful.

This is why the manuscript uses the term conditional visibility. Institutional voice is not fully manageable because it enters a platform environment shaped by user uptake, journalistic selection, and algorithmic circulation. It is also not irrelevant. It can become consequential when attached to a structurally charged event. The contribution is to specify the conditions under which discourse becomes visible, not to claim a universal mechanism of algorithmic amplification.

### 5.3. Implications for sport organizations

For sport organizations, the results caution against a simple speed rule. Rapid response is important because delayed communication may miss the immediate attention window. However, speed alone does not determine the scale or valence of attention. After title wins, expressive or legacy-oriented communication may be appropriate because celebration is expected, shareable, and institutionally legitimate. After humiliating defeats or reversals, the present evidence is weaker; crisis-minimalist communication may become visible, but this pathway requires further testing and should not be treated as a general prescription.

The practical implication is risk-sensitive calibration rather than deterministic control. Clubs cannot reliably create high attention after a low-stakes event through stylistic creativity alone, and they cannot reliably suppress attention after a high-stakes shock through response speed alone. Communication teams should therefore assess the event configuration before choosing tone, timing, and message form.

## 6. Limitations and future research

The study has three main limitations. First, the sample consists of 25 theoretically selected UEFA Champions League cases. This design is suitable for fsQCA but does not support population-level estimation. Second, News_Index is a log-normalized Raw_Mentions-derived media-visibility proxy. It improves transparency compared with an opaque composite, but it does not capture every form of digital attention, such as quote-post networks, meme diffusion, multilingual fan discourse, or platform-internal ranking. Third, High_TemplateDeviation is based on a structured author audit. The structured audit improves transparency through Deviation_Type, Deviation_Evidence, Coding_Confidence, Robustness_Flag, and strict robustness checks, but future research should add independent coders, intercoder reliability statistics, and more fine-grained linguistic measures.

Future studies can extend the design in two directions. A temporal extension could model burst, decay, and secondary-wave dynamics. A comparative extension could examine domestic leagues, non-European competitions, or clubs with different fan bases and media ecologies. These extensions would help distinguish event-driven attention from organization-specific reputation effects.

## 7. Conclusion

This study examined why some UEFA Champions League post-match institutional communications become associated with high collective attention while others remain comparatively peripheral. Using a theoretically selected sample of 25 focal club-event observations and fsQCA, the study shows that High_News is not explained by any single condition in isolation. The strongest evidence supports a stable high-stakes legacy pathway: non-delayed institutional communication, high match stakes, low focal-outcome shock, and observable trophy/legacy-oriented template deviation.

The analysis is deliberately conservative. It separates response latency from communication form, treats News_Index as a Raw_Mentions-derived proxy rather than a comprehensive attention index, and distinguishes a stable pathway from a sensitivity-dependent crisis-minimalism pathway. The findings therefore provide reproducible configurational evidence within a bounded UEFA Champions League sample, not population-level claims about all football matches or internal platform algorithms.

## Supporting information

S1 File Supporting information archive. This ZIP archive contains the 25-case analytical dataset and calibrated QCA input files, the case-selection audit, the truth table for High_News, robustness-check outputs, reproduced necessity and solution metrics, the reproduction code, and a supplementary information document describing these materials.
(ZIP)
