# Supplementary material for: Beyond speed and strategy: A configurational analysis of collective attention to post-match institutional discourse in the UEFA Champions League
Source: PLoS One. 2026 Jul 28;21(7):e0354175. doi: 10.1371/journal.pone.0354175 (PMC13411929; doi:10.1371/journal.pone.0354175)
Supplement: S1 File — This ZIP archive contains the 25-case analytical dataset and calibrated QCA input files, the case-selection audit, the truth table for High_News, robustness-check outputs, reproduced necessity and solution metrics, the reproduction code, and a supplementary information document describing these materials. (ZIP) [file pone.0354175.s001.zip › Supporting Information/PONE-D-26-04408_Supplementary_Information.docx]

# Supplementary Information

Beyond speed and strategy: A configurational analysis of collective attention to post-match institutional discourse in the UEFA Champions League

## S1. File inventory and reproducibility statement

The revision supplies five supporting files. S1 Dataset contains the revised 25-case analytical dataset, raw and calibrated indicators, source-trace fields, and coding evidence. S2 Case Selection Audit reports the broader UEFA Champions League match audit used to identify retained and non-retained candidates. S3 Truth Table reports observed truth-table rows. S4 Robustness Checks reports alternative calibration and coding-sensitivity checks. S5 Reproduction Code reproduces News_Index, calibrated sets, truth-table rows, solution metrics, and R1-R9 robustness outputs.

All values in the revised manuscript are recalculated from S1 Dataset and can be regenerated with S5 Reproduction Code. The code avoids hidden spreadsheet formulas and can be executed in a local Python environment with pandas and numpy installed.

## S2. Variable dictionary

### Table S1. Variable dictionary.

| **Variable** | **Description** |
| --- | --- |
| Raw_Mentions | Retrievable media mentions for the focal club-event observation |
| News_Index | Log-normalized 0-100 media-visibility proxy derived from Raw_Mentions |
| High_News | Fuzzy membership in high collective attention |
| Raw_Delay | Minutes from final whistle to first substantive institutional post |
| High_Delay | Fuzzy membership in delayed response |
| High_Shock | Fuzzy membership in high outcome shock |
| High_Stakes | Fuzzy membership in high match stakes |
| Deviation_Type | Audited communication-form category |
| High_TemplateDeviation | Fuzzy membership in strong template deviation |
| Coding_Confidence | Evidence-quality label for template-deviation coding |
| Robustness_Flag | Flag identifying main, sensitivity, strict-recoding, or alternative-specification cases |

## S3. Case-selection criteria

The revised manuscript does not use “iconic” as an undefined category. A retained case had to satisfy at least two criteria: structural salience, clearly identifiable event narrative, identifiable official-post timing, and retrievable Raw_Mentions. S2 Table reports the retained and excluded candidates.

## S4. Calibration formula

For quantitative variables, membership scores follow direct piecewise linear calibration. Values at or above the full-membership anchor receive 1.0; values at or below the full-non-membership anchor receive 0.0; values between the crossover and either endpoint are linearly interpolated. The exact function is provided in S5 Reproduction Code.

## S5. Baseline solution summary

### Table S2. Baseline solution summary.

| **Pathway** | **Formula** | **Consistency** | **Raw coverage** |
| --- | --- | --- | --- |
| Stable high-stakes legacy pathway | ~High_Delay x ~High_Shock x High_Stakes x High_TemplateDeviation | 0.912 | 0.342 |
| Sensitivity-dependent crisis-minimalism pathway | ~High_Delay x High_Shock x High_Stakes x High_TemplateDeviation | 1.000 | 0.045 |
| Overall solution | Union | 0.921 | 0.387 |

## S6. Notes on interpretation

The stable pathway carries the main interpretive weight. The crisis-minimalism pathway is retained in the baseline solution but should be interpreted cautiously because it is removed under conservative template-deviation recoding. This is reported in the manuscript and S4 Robustness Checks.
